# Estimating Rupture Risk of Intracranial Aneurysms: What We Know, What We Do Not Know, and What We Need

**DOI:** 10.1161/STROKEAHA.125.054863

**Published:** 2026-04-08

**Authors:** Samuel Hall, Frederick Ewbank, Benjamin Gaastra, Nazrul Islam, Jacqueline Birks, Diederik Bulters, on behalf of the Risk of Aneurysm Rupture (ROAR) Study Group, Giles Critchley

**Affiliations:** 1 Department of Neurosurgery, University Hospitals Southampton NHS Foundation Trust, United Kingdom (S.H., F.E., B.G., D.B.).; 2 Neurosciences, Clinical and Experimental Sciences, Faculty of Medicine (F.E., B.G., D.B.), University of Southampton, United Kingdom.; 3 Population Sciences, Faculty of Medicine (N.I.), University of Southampton, United Kingdom.; 4 Centre for Statistics in Medicine, Medical Sciences Division, University of Oxford, United Kingdom (J.B.).

**Keywords:** bias, cohort studies, intracranial aneurysm, risk assessment, risk factors, subarachnoid hemorrhage, survival analysis

## Abstract

Management of unruptured intracranial aneurysms requires balancing the risk of aneurysm rupture against the risk of procedural complications. Estimates of rupture risk stem from a few landmark natural history studies whose findings differ substantially, creating uncertainty for clinical decision-making. This review appraises these studies, highlighting areas of agreement and contradiction to inform future directions. Across studies, short-term rupture risk is low and increases with aneurysm size. The magnitude of risk varies (0.20%–1.85% at 1 year). These discrepancies likely arise from methodological challenges inherent to natural history research, including selection, crossover, incomplete follow-up, and regional variation. The effects of these factors are difficult to disentangle due to confounding. Rupture risk is highest in Finnish studies, followed by Japanese, then other international cohorts. This geographic pattern is reversed for the treatment rate. Rupture risk also shows a strong inverse relationship with treatment rate (*P*=0.008, R^2^=0.79). This makes it impossible to know whether rupture risk reflects treatment or geography, and which estimates apply clinically. Studies have short follow-up (mean 2.8 years) and require substantial extrapolation to estimate lifetime risk (mean age at diagnosis 42–66 years). Small differences in short-term estimates produce large variations in long-term projections. Moreover, the underlying assumption that risk remains constant with time has not been formally evaluated. Half of the data sets are consistent with this, but half suggest it declines with time. The effects of key aneurysm rupture predictors vary between studies. This includes age, sex, hypertension, smoking, prior subarachnoid hemorrhage, family history of intracranial aneurysms, and aneurysm location, multiplicity, and size thresholds. It is unclear whether this reflects regional variation, overfitting, or other factors. Meta-analyses are most representative, but remain constrained by limitations of contributing data sets. Larger multicenter studies with longer follow-up, fewer losses, and deeper phenotyping are still needed, despite their practical challenges.

Aneurysms are local dilatations of blood vessels that arise from structural weakening of the vessel wall. They can occur throughout the body, including intracranially on the arteries of the circle of Willis and its branches. Intracranial aneurysms (IAs) characteristically arise in thin-walled arteries at bifurcation points where hemodynamic stress is greatest, whereas aneurysms of the aorta and peripheral circulation usually develop in thick-walled elastic arteries affected by atherosclerotic changes.

IAs occur in ≈3%^1^ of adults. Most remain unruptured (unruptured IA [UIA]) and either go undetected or are discovered incidentally on neuroimaging. Their rupture causes subarachnoid hemorrhage (SAH), which has a mortality of >50%.^2^ However, preventive treatment of UIA also carries serious risks of bleeding and stroke and is therefore only offered to patients at sufficiently high risk of rupture.

Ideally, UIA treatment decisions would be informed by randomized controlled trials comparing intervention with conservative management. However, prior attempts were unsuccessful and only recruited 80 out of a planned 2000 cases.^3^ Although recent randomized controlled trials have compared treatment modalities^4,5^ a direct comparison with observation is unlikely to be feasible. Moreover, randomized controlled trials are designed to determine a single best treatment strategy for the enrolled patient population, whereas patients with UIA are a heterogeneous population, and as such, management decisions should probably be individualized, accounting for the unique rupture risk profile of each patient.

This is in keeping with the American Heart Association and American Stroke Association^6^ and European Stroke Organisation^7^ guidelines, which both recommend accounting for age/life expectancy, prior SAH, family history of IA, IA location, and IA size when deciding whether to perform preventive repair (Table S1).

The natural history data needed to guide these decisions come from a limited number of large observational cohort studies. The landmark ISUIA (International Study of Unruptured Intracranial Aneurysms) was published in 2003.^8^ This and subsequent studies were synthesized in the 2014 Population, Hypertension, Age, Size, Earlier SAH, Site (PHASES) pooled analysis.^9^ Some of these studies individually received critical correspondence at the time of their publication.^10–13^ However, they represent the best attempts to address this issue and provide the most robust data for clinical decision-making. Although many have featured in broad reviews of risk factors for IA and their rupture,^14,15^ there has been no focused appraisal of the methodological strengths and weaknesses of these longitudinal cohorts. This is needed given there is no consensus on which studies are representative of patients seen clinically, and therefore, which should guide treatment decisions.

When designing the ROAR study (Risk of Aneurysm Rupture),^16^ we systematically evaluated the cohorts that inform UIA rupture risk estimates. This article presents a critical appraisal of those studies, highlighting key methodological challenges in estimating long-term rupture risk and identifying opportunities for improvement in future research.

## What We Know—Published Studies

We reviewed the studies in PHASES and have performed a systematic literature search for any additional studies since the publication of PHASES. The studies are summarized in Table 1; their populations in Table 2; and their rates of selection, treatment, crossover, and follow-up in Table 3. Whereas the tables provide a structured comparison between studies, we use the text to highlight their most notable features.

**Table 1.**
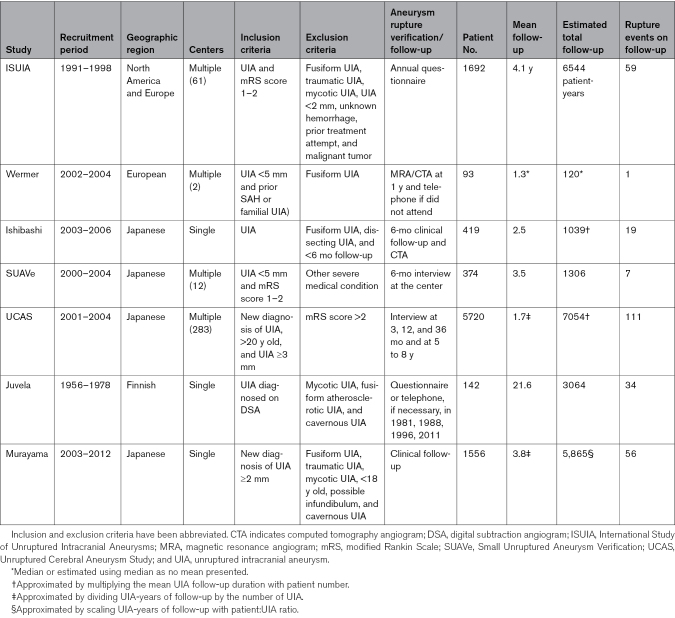
Summary of Studies

**Table 2.**
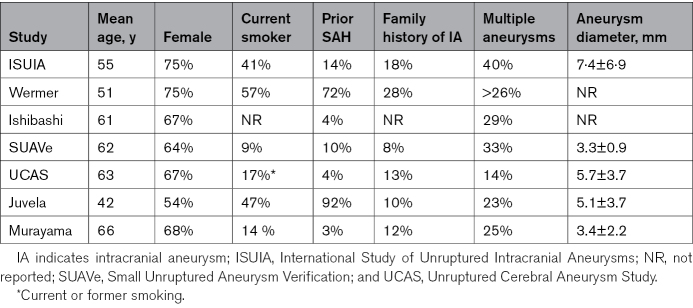
Summary of Study Populations

**Table 3.**
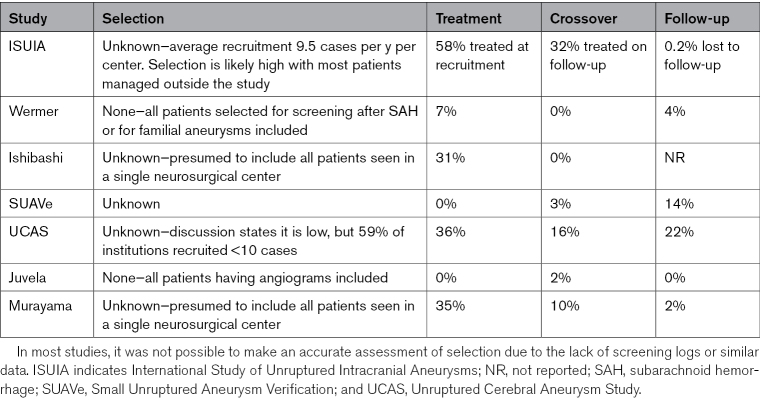
Summary of Study Selection, Treatment, Crossover, and Follow-Up Bias

### Evidence Before PHASES

#### ISUIA (2003)

This prospective observational study followed 1692 patients from 61 centers for 4.1 years. Patients were recruited through the 1990s in the United States and Europe. The study included a broad range of patients and UIA.^8^

The main criticism was the study’s pronounced selection bias due to treatment. Of the 4060 patients initially evaluated, 71% were treated (2368 at recruitment and 534 during follow-up). The true selection bias was probably far greater because it is unknown how many patients were screened but not enrolled. Many high-volume centers contributed few cases (eg, Helsinki, Miami, Mannheim, and Berlin each recruited <10, including treated cases). Even the top recruiting center (Mayo, 293 cases) reported far fewer than expected (our own unit would expect to see over 900 new cases over the same timeframe), suggesting most UIAs were managed outside the study.

This selection bias could leave a nonrandom, lower-risk subset for observation. Although ISUIA stratified risk by UIA size and location, it did not adjust for morphology, having multiple IA (multiplicity), or family history of IA or SAH, factors likely to influence treatment and rupture risk.

Further complicating the interpretation is the 534 (32%) observed patients who crossed over to treatment. Reasons for crossover are not stated, but many likely had interval growth or morphological changes, which may be markers of increased rupture risk.^17–19^ Their removal from observation probably deflated rupture estimates. However, there may be additional reasons for crossover, as 32% far exceeds reported growth rates. It is unknown what the reasons for crossover were and how they influenced findings.

The study found an exceedingly low 5-year rupture risk—particularly among anterior circulation UIA <7 mm, in whom no patient without prior SAH suffered a rupture.

These findings shaped clinical practice but also sparked controversy. It remains the only large study of a Western population, but it also remains uncertain to what degree selection bias was responsible for the low rupture rates observed.

#### Wermer (2006)

This Dutch cohort followed 93 patients with UIA, identified through screening of individuals with a personal history of SAH or family history of SAH or UIA, for a median of 1.3 years. The study is of high quality, but with small numbers and short follow-up, it only observed 1 rupture event.^20^

Study power is determined not directly by sample size but indirectly through outcome events. Therefore, while sample size is an issue for all studies, in the case of Wermer, the sample size is so small and the follow-up so short that it provides insufficient data to influence our understanding of rupture risk.

#### Ishibashi (2009)

This single-center series followed 419 Japanese patients for 2.5 years. Although the study accepted UIA of all sizes, clinical practice was for UIA >5 mm to be treated, and the series is predominantly comprised of small UIA. ^21^

Its main limitation is how applicable the observed rupture rates are to non-Japanese populations, given that the incidence of aneurysmal SAH in Japan is reported to be higher than the rest of the world.^22^

All patients seen in this unit were considered for the study. The overall rate of UIA treatment (31%) was a lot lower than in ISUIA (71%). These were all treatments before entry into the study (and suggested to be limited largely to UIA >5 mm). Of those followed up, no patients were treated and crossed over.

This study demonstrated a higher UIA rupture risk than ISUIA.

Although this study suggests that UIA rupture risk may be higher than ISUIA, being a much smaller single-center series, it was not definitive, and the results require validation. Moreover, the reasons why the UIA rupture risk was higher were not clear, including whether this was due to the lower treatment rates or an inherently higher rupture risk in Japan. Hence, it was unclear whether the higher UIA rupture risk applied only to patients in Japan or also to patients in the rest of the world.

#### Small Unruptured Aneurysm Verification (2010)

The SUAVe (Small Unruptured Aneurysm Verification) followed 374 patients from 12 centers with UIA <5 mm diameter for 3.5 years in response to a national policy in Japan to not treat UIA this size.^23^

It did not specifically record treatments performed before recruitment, but implies that few occurred, and states none occurred immediately after recruitment. This low treatment rate in a clearly defined group of UIA is a strength. All patients underwent surveillance imaging, and all but 27 completed it. Only 10 (2.7%) crossed over to treatment due to growth. This is far fewer than ISUIA (31%) and is explained by a stringent definition for growth (≥2 mm change).

However, it is worth noting that if treatment is low, but also carefully targeted, as in this study, the effects of crossover can still be significant. UIA growth can be difficult to adjudicate, and small apparent changes in size may be artifactual due to scan variability or, even if real, may not necessarily translate into an increased rupture risk. Targeted treatment of cases of definite or substantial growth may introduce similar or greater bias than liberal treatment with less stringent thresholds for defining growth.

The study showed a higher rate of rupture than ISUIA and validated the findings of Ishibashi. However, like Ishibashi, it is unclear if this difference is due to where it was conducted or due to low selection bias.

#### Unruptured Cerebral Aneurysm Study (2012)

The UCAS (Unruptured Cerebral Aneurysm Study) followed 5720 patients recruited at 283 sites in Japan for 1.7 years. It is the largest UIA study to date and comprises nearly 70% of cases in the literature. Due to its short follow-up duration, the estimated total follow-up is only modestly larger than the other studies and represents 28% of available data in terms of patient-years of follow-up.^24^

UCAS provides more methodological detail than most studies and is one of the few studies that addresses selection in the cohort clearly, stating they did not collect data on patients not enrolled in the study. The study had a slightly higher rate of treatment than the other Japanese studies (48%).

The study again showed a higher rupture risk than ISUIA, although it was slightly lower than Ishibashi and SUAVe.

The study clearly validates the findings of Ishibashi and SUAVe and provides excellent data to inform decisions for Japanese patients. The slightly higher treatment rate than in those studies could explain the slightly lower reported UIA rupture risk than in Ishibashi and SUAVe. However, the UIA rupture risk of the Japanese studies still seems to cluster together, leaving it uncertain if the results are applicable elsewhere.

#### Juvela (2013)

This study identified 142 patients from records of angiograms performed between 1956 and 1978 and followed them intermittently (around 1981, 1988, 1996, and 2011) by questionnaire or by telephone if necessary. Angiograms were mostly performed for SAH from another IA. It was performed at a time when ruptured, but not UIA, were treated. Finland during this period also had extremely high smoking rates. The study was one of the smallest studies by patient number, but its unique long-term follow-up resulted in a large number of patient-years for analysis.^25^

Juvela also stands out as the only study almost free from selection bias. All patients were included in the study; virtually none were treated during follow-up (3 patients were treated after ≈25 years), and none were lost to follow-up.

Rupture occurred in 24% of patients. This is in stark contrast to other studies and challenges extrapolations of their short-term data. ISUIA suggested 0% 5-year rupture risk for small anterior UIA, whereas Juvela found 30% lifetime rupture risk.^26^

There are several possible explanations for this: the Finnish population may have an inherently higher UIA rupture risk; the specific population studied may have had a higher risk; or other studies may have underestimated risk due to treatment-related selection bias.

Finland (like Japan) has historically reported a much higher incidence of SAH than other countries.^27^ This has commonly been invoked to explain the high UIA rupture rate in Juvela. However, more recent registry data challenge this assumption, showing that the incidence of SAH in Finland is similar to that of other Nordic countries.^28^

If Finnish UIA rupture risk does differ, it could reflect genetic or environmental factors (or both). Although understanding of the genetic predisposition to IA has improved,^29^ no specific Finnish (or Japanese) genetic variant has been identified. Of possible environmental factors, smoking would be the best candidate. 47% of study participants were current smokers at baseline. If the effect of smoking on UIA rupture was large, this could account for the high rupture rate in this study, and would be consistent with the observation that SAH in Finland has been declining at roughly the same rate as smoking.^28^ However, the magnitude of the effect of smoking on UIA rupture risk is unclear from the literature (see section What We Do Not Know—Smoking).

Another distinctive feature of this cohort, which is neither environmental nor genetic, is that 92% had a prior history of SAH from another aneurysm. Patients with prior SAH are suspected to be at higher risk of UIA rupture. This may have increased the observed UIA rupture risk in Juvela, although the natural history literature is also unclear about the magnitude of the effect of prior SAH (see section What We Do Not Know—Prior SAH).

Ultimately, although multiple plausible explanations exist for the high UIA rupture rate observed in Juvela, we cannot determine whether this arises from the Finnish population, the high prevalence of prior SAH, the absence of treatment, or something else. This makes it difficult to determine how Juvela should contribute to estimating UIA rupture risk outside of Finland.

### PHASES Pooled Analysis

These 6 studies underwent a pooled analysis of 8382 patients with 29 166 years of follow-up to estimate 5-year rupture risk using 6 covariates (PHASES). It is our best composite estimate, but it is not without limitations.^9^

Critically, the cohorts were from disparate populations with no overlap (Finnish, Japanese, or neither) and different rupture rates. Meta-analysis assumes low heterogeneity.^30^ No assessment of heterogeneity is presented, but it is likely to have been high. A new variable (“ethnicity”—Finnish, Japanese, neither) was added to reconcile differences between studies, assuming they stem from population rather than methodological variation. This remains unproven. If the differences are methodological, the inclusion of ethnicity would markedly distort risk estimates.

Using ethnicity also risks obscuring environmental confounders. For instance, if Finland’s rupture risk stems from smoking, ethnicity as a covariate could mask smoking’s effect. PHASES notably did not find smoking predictive of UIA rupture.

There were additional minor issues with the handling of covariates. Smoking and hypertension were missing from one study and were imputed for all patients.^21^ The range of covariates was limited and likely based on availability in underlying studies, which in turn is limited by the difficulties of collecting deep phenotypic data in large populations. Many other plausible risk factors exist, and it is likely that the 6 in PHASES only explain a small fraction of the variance in risk. The PHASES model could therefore almost certainly be further personalized using a wider range of UIA rupture predictors, although these additional datapoints may be progressively more difficult to collect.

### Evidence Published Since PHASES

We searched PubMed using the same search terms as PHASES (Table S2) for the period from the date of the PHASES search to the date of our search (July 26, 2013–June 11, 2025). Studies were selected that: (1) included ≥ 50 patients with UIA, (2) studied the natural course of UIA and studied risk factors for IA rupture, (3) used a prospective study design, and (4) had IA rupture (aneurysmal SAH) as an outcome. In total, 1187 studies underwent independent review of the title and abstract by S.H. and J.B., 20 underwent full text review, and 9 met inclusion criteria for PHASES (Table S3).

Most were small, short follow-up studies with few rupture events.^18,31–38^ Some of these individual studies are included in the most recent European Stroke Organisation guidance,^7^ which pools data from 9 studies. These 9 studies report 1-year rupture risks ranging from 0.2% to 1.85% and yield a pooled 1-year rupture risk estimate of 0.81% (95% CI, 0.61–1.05).

The only large study was from Murayama, reporting 2252 patients from a single-center database in Japan.^35^ Its title, Prospective 10-year Cohort Study, refers to the recruitment period (2003–2012), not the duration of follow-up (mean 3.2 years). It provides an important additional 5865 years of follow-up and found a similar rate of rupture to UCAS.

Given that it was performed in a Japanese population and had similar rates of treatment and UIA rupture to UCAS, it validates this study but does not resolve the outstanding uncertainties from PHASES.

## What We Do Not Know—Outstanding Uncertainties

### Overall Rupture Risk

**Figure 1.**
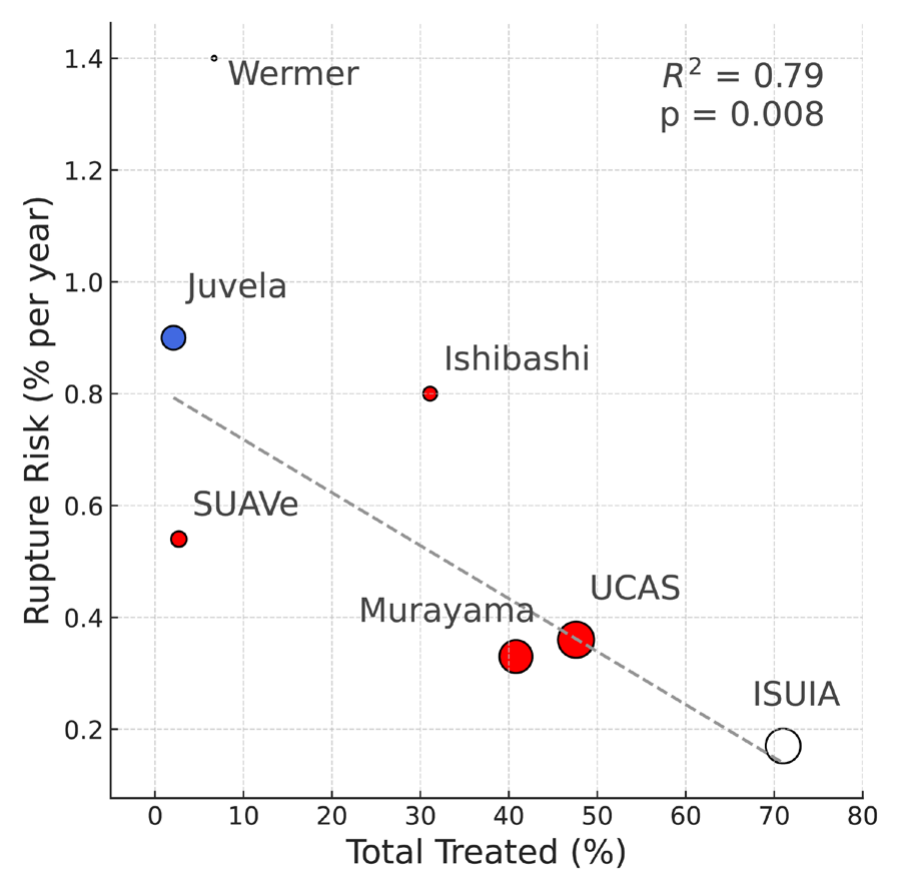
**The relationship between observed rupture risk for small unruptured intracranial aneurysms (UIA) and treatment rates in studies.** This includes treatments at baseline and on follow-up. The size of points is scaled to the study size (number of patient-years of follow-up). Finnish studies are in blue, Japanese in red, and non-Finnish non-Japanese in white. The gray dotted line is the weighted linear regression line (weighted by study size). See Table 2 for details of studies and Figure S1 for similar depictions for those treated at baseline and those treated on follow-up. ISUIA indicates International Study of Unruptured Intracranial Aneurysms; SUAVe, Small Unruptured Aneurysm Verification; and UCAS, Unruptured Cerebral Aneurysm Study.

As discussed, estimates of UIA rupture risk vary widely (Table S4). Figure 1 illustrates the strong inverse association between treatment rate and rupture risk (total treatment weighted *β*=−0.009, *P*=0.008, *R*^2^=0.79, subgroups shown in Figure S1), as well as the relationship between treatment rate and population. This demonstrates how these effects cannot be disentangled to establish which rupture risk estimates are clinically relevant.

### Long-Term Risk

Except for the 142 patients in Juvela, studies have a short mean follow-up between 1.7 and 4.1 years (mean follow-up of all patients 2.8 years). Typical patients presenting in their 50s have life expectancies of over 30 years. Extrapolation of short-term estimates over lifetimes amplifies uncertainty as even small differences can become important. For example, CIs for PHASES estimates may seem small (0.1%–1.5% 5-year risk for the lowest risk group) but become clinically significant if extrapolated (0.6%–9% 30-year risk).

**Figure 2.**
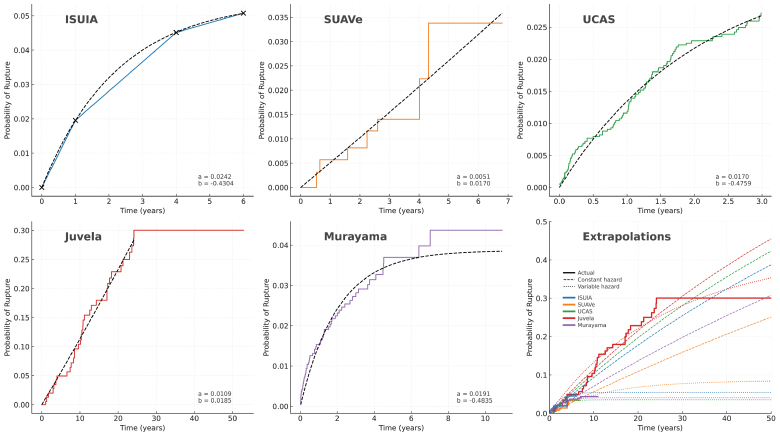
**Probability of rupture plots.** Survival plots from each study providing survival data have been digitized and cumulative Gompertz distribution curves fitted (black dashed lines) to evaluate if the instantaneous unruptured intracranial aneurysm rupture risk (hazard) is constant over time. The studies show the full range of possibilities from constant hazard to clearly reducing hazard. This makes extrapolation misleading, as shown in the final figure, where dashed lines represent long-term extrapolation assuming constant hazard (dashed line) or variable hazard (dotted line). This demonstrates a >10-fold difference in long-term rupture risk estimates. Survival plots were digitized using WebPlotDigitiser and expressed as the probability of rupture. For ISUIA (International Study of Unruptured Intracranial Aneurysms), data were only presented for subgroups, and a weighted average for each time point was used. Cumulative Gompertz distribution curves, modeling the estimated probability of UIA rupture over time based on observed data, were optimized using nonlinear least squares regression. For Juvela, a good fit could not be achieved with the full data set, which was therefore limited to the first 25 years of follow-up. Estimated model parameters (*a* and *b*) are shown in the **bottom-right** of each subplot. The extrapolations in the final plot were made using a constant hazard (exponential function) or variable hazard (Weibull function). ISUIA indicates International Study of Unruptured Intracranial Aneurysms; SUAVe, Small Unruptured Aneurysm Verification; and UCAS, Unruptured Cerebral Aneurysm Study.

Such extrapolation also depends on the assumption that instantaneous risk (hazard) remains constant over time. However, it is not known if this assumption is correct. There are no formal analyses of this, although 5 studies provide time-to-event plots (Figure 2). Data from 2 studies with short-term follow-up suggest a roughly constant hazard (SUAVe and UCAS), whereas 2 others suggest the hazard decreases with time (ISUIA and Murayama).

Juvela provides the only data to indicate how rupture risk changes over long periods of time. The cumulative incidence of rupture was approximately linear for 25 years, suggesting a near constant hazard, after which there was a sudden plateau, and no further events were observed. Interpretation is difficult because CIs of the Kaplan-Meier estimates would have been wide, particularly in the later years, as the number of patients in follow-up reduced (72 patients at 20 years).

From this data, we cannot determine the long-term behavior. This makes any attempt to project long-term risk potentially misleading. The extrapolations in Figure 2 first illustrate how any small differences in short-term rupture risk lead to large variations in long-term estimates when a constant hazard is assumed. However, it also shows that uncertainty over whether rupture risk is constant or time-varying generates even larger differences in long-term UIA rupture risk. Combining the uncertainty over short-term risk and how risk varies over time, long-term estimates vary over 10-fold.

### Effect of Individual Risk Factors

#### UIA Size

Although UIA size is clearly associated with rupture risk, there are clinically important inconsistencies relating to the size thresholds, particularly for small UIA. Specifically, it is unclear if all UIA <7 mm carry a similar risk of rupture or if the risk of rupture differs between those 5-6 mm and those <5 mm. Despite stark differences between ISUIA and Juvela, both found no thresholds to distinguish risk within the small (<7 mm) group. UCAS also found no difference between UIA 3 to 4 mm and 5 to 6 mm in diameter. Contrary to this, Ishibashi, SUAVe, and Murayama all found a difference between these groups. With half of the studies showing a difference and half not, it is unclear if all UIA <7 mm should be managed the same or if treatment decisions should be further personalized based on size within this group.

#### UIA Location

UCAS and ISUIA were the largest studies contributing to the PHASES score. There are notable similarities and differences in how UIA location influences rupture risk in these studies.

At the time of publication, it was highly controversial that ISUIA grouped posterior communicating UIA with posterior circulation UIA. Although the names may seem similar, posterior communicating UIA arise from the internal carotid artery and are in the anterior circulation and not on the vertebrobasilar system of the posterior circulation. Moving them from the anterior circulation to the posterior circulation appeared to be fitting models to the data and was responsible for why there were no rupture events in the anterior circulation group. However, UCAS also found posterior communicating UIA high-risk, validating grouping them with UIA of the posterior circulation.

The rupture risk associated with other UIA locations varies significantly across studies. UCAS identified the anterior communicating artery (ACom) as the highest-risk site, while ISUIA grouped anterior communicating artery UIA with other low-risk anterior circulation UIA. This creates a major discrepancy: clinicians may either follow ISUIA and consider anterior communicating artery UIA low-risk or follow PHASES, which is based heavily on UCAS, and manage them as high-risk.

Other inconsistencies include basilar tip UIA, which were among the highest risk in ISUIA but had rupture rates similar to middle cerebral artery UIA in UCAS (which were low risk in ISUIA). Juvela also contradicts ISUIA, identifying only the anterior communicating artery location as a significant risk factor. Importantly, none of these studies performed internal validation to assess overfitting, nor did they externally validate their models. Thus, observed differences may reflect true risk, overfitting, or methodology.

#### Age

PHASES concluded age >70 years to be a risk factor, contradicting SUAVe and Juvela, which found a higher risk in patients <50 years old. The remaining studies observed no associations between age and the risk of UIA rupture.

The true relationship between age and rupture risk remains unknown. A common belief is that UIA stabilizes over time. This means older individuals, whose UIA may have been present for longer, should have a lower rupture risk after diagnosis. This was not the case in PHASES.

A possible explanation for why some individual studies suggested UIA in older patients was at lower risk, whereas the PHASES meta-analysis indicated higher risk, lies in how the populations were pooled. For example, ISUIA recruited younger patients on average (mean age 55 years) compared with the Japanese studies (61–62 years).^17,21,23,24^ If ISUIA underestimated rupture risk because of selection bias and coincidentally enrolled a younger population, this could create the appearance in the pooled analysis that older age itself was a risk factor.

The apparent link between older age and higher rupture risk in PHASES may also reflect residual confounding, as comorbidities such as hypertension that are more common in older patients were inconsistently captured across cohorts, and could not be fully disentangled from the effects of age even when recorded.

#### Prior SAH

Although prior SAH is commonly considered a risk factor for rupture, this was only observed in ISUIA for UIA <7 mm and in the Ishibashi and Murayama studies. It was not seen in the remainder of the ISUIA cohort with larger UIA, nor in the Juvela, SUAVe, or UCAS cohorts. In PHASES, prior SAH emerged as a relatively weak predictor of UIA rupture, and the use of the ethnicity variable may have attenuated its effect, given that 92% of patients in the Juvela cohort had a prior SAH.

#### Hypertension

Only SUAVe and PHASES reported a significant association between hypertension and rupture. Other studies showed borderline results (*P*=0.06 in Juvela, *P*=0.08 in UCAS), and Murayama found none. Hypertension is likely a risk factor, but the effect size may be small or mitigated by blood pressure control after diagnosis.

#### Smoking

Despite strong external evidence linking smoking to SAH, including epidemiological,^39^ temporal,^22^ and twin studies^40^ it was not significant in PHASES or Murayama. Smoking is of course, intrinsically difficult to study due to a strong social desirability bias when obtaining responses from patients. However, there are other possible reasons why no relationship between smoking and SAH was found.

Collinearity between smoking and ethnicity would explain the absence of a relationship. This is supported by the observation that smoking was significantly associated with univariable but not multivariable analysis in PHASES. However, we found no association between smoking prevalence and rupture risk at the study level in the reviewed studies (weighted *β*=0.005, *P*=0.675, *R*^2^=0.07). An alternative explanation is that the binary categorization in PHASES (ever smoker/never smoker) may have diluted risk by inclusion of ex-smokers in the smoking group. However, in Murayama, ex-smokers were not at lower risk when compared with smokers. The final explanation would be that the risk was reduced by smoking cessation during follow-up. However, compliance with this is generally poor, and Juvela and Murayama, which were some of the few studies that recorded smoking on follow-up, noted only 11 and 18% stopped.

#### Family History

Patients with a family history of IA or SAH have always been suspected by clinicians and patients to be at higher risk of rupture. Some recent but limited data support this suspicion.^41^ However, family history of IA or SAH was not a significant predictor of UIA rupture in any of the individual studies and was not reported in PHASES. Therefore, its influence on rupture risk remains unknown.

#### Multiplicity

One study^23^ found increased rupture risk with IA multiplicity, but 3 did not.^21,24,25^ Descriptions of how multiplicity was managed in the analysis are limited. One might expect patient-level and aneurysm-level influences on risk. These should be analyzed using a multilevel model. Only PHASES clearly states its methodology. This used a single-level model performed at the patient level using the largest UIA for UIA characteristics. This type of model assumes independence between all IAs, which is unlikely to be the case. No other studies describe the details of their modeling, with the implication that it was a single-level analysis. Some may have analyzed the aneurysm rather than the patient level, although it is not possible to know. There is no discussion whether clinically the implication of this is that in the setting of multiple UIAs, rupture risk is simply additive, or determined by the single largest UIA, or if the relationship is more complex. There is also no discussion of the obvious collinearity and interactions that exist between patients with multiple IAs and those with prior SAH. Taken together, we do not know if multiple UIAs carry additional risk or how to calculate the combined risks in patients with multiple UIAs.

#### Sex

One study found evidence to suggest females have a higher risk of UIA rupture (*P*=0.05),^24^ whereas 2 others did not.^25,35^ PHASES found no relationship with sex, despite a more recent analysis utilizing many of the same cohorts finding that females had higher UIA rupture risk.^42^

#### Daughter Sac

Two studies found an increased UIA rupture risk, albeit with different effect sizes (hazard ratio, 1.63 and 11.10). ISUIA recorded data on aneurysm morphology but did not report it as a risk factor, suggesting it was not significant.

**Figure 3:**
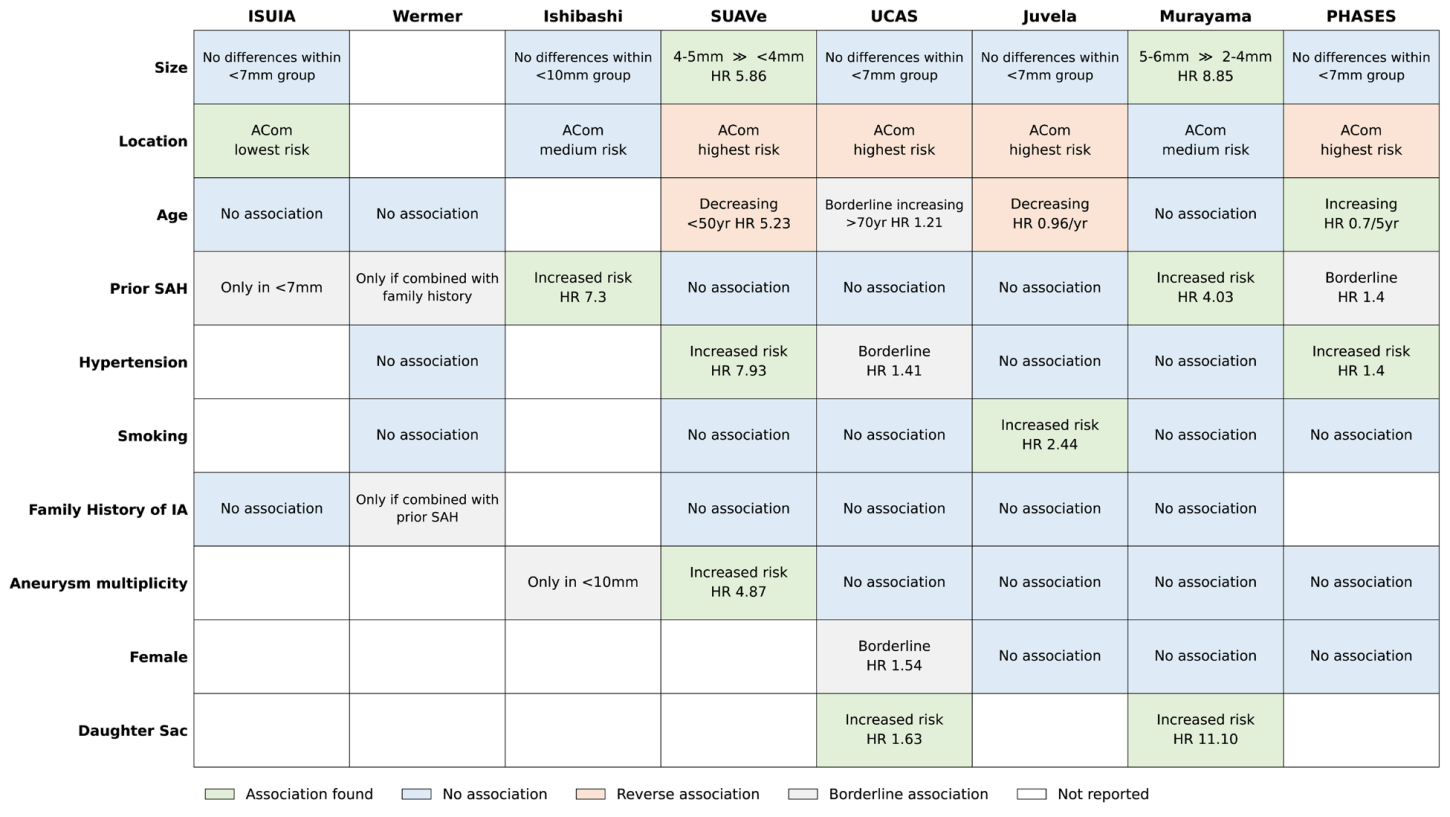
. **Proposed risk factors for unruptured intracranial aneurysm (UIA) rupture.** There are contradictions in the supporting evidence for each one. While all studies find size to predict rupture, they vary in whether it can stratify small aneurysms (<7 mm) further into higher- and lower-risk small aneurysms. The anterior communicating artery (ACom) location is shown as one example of variation in risk with location. All other locations show variation between studies. HR indicates hazard ratio; IA, intracranial aneurysm; ISUIA, International Study of Unruptured Intracranial Aneurysms; PHASES, Population, Hypertension, Age, Size, Earlier SAH, Site; SAH, subarachnoid hemorrhage; SUAVe, Small Unruptured Aneurysm Verification; and UCAS, Unruptured Cerebral Aneurysm Study.

Taken together, there is inconsistency in the association of UIA rupture with every proposed risk factor (Figure 3).

## What We Need—Considerations For Future Studies

To plan future studies, we assessed the risks of common types of bias in existing studies and examined how these could be minimized within the ROAR study. These are presented in Table S5. The following are areas in need of particular consideration:

### Population

Future studies must better define and diversify their population. Ideally, this would represent the full spectrum of patients with UIA and not be limited to high-risk groups like those with a family or personal history of SAH or IA. Additionally, they would include patients from around the world, or at least not be from solely Finnish or Japanese groups. They will also need to better understand why there are such large variations in UIA and patient characteristics between studies (Table 2; female 54%–75%, smoking 14%–57%, prior SAH 3%–92%, family history of IA or SAH 8%–28%, multiple IA 14%–40%).

### Selection Bias

Only 2 studies reported how many eligible patients were excluded, of which only one gives reasons why.^23,24^ Some, like ISUIA, probably only recruited a tiny fraction of eligible patients. Future studies must include comprehensive screening logs, including reasons for exclusions and detailing the characteristics of these patients. Ideally, all patients would be included, although this is problematic due to requirements for patient consent to follow-up. Alternative approaches may therefore be needed. For example, in the UK national databases of hospital admissions and deaths can be used to track SAH events. Permission can be obtained from the national Confidentiality Advisory Group to use these for research without patient consent. All households in England were written to in 2014 to explain this and how to opt out, and patients continue to be able to opt out of digital research using the National Data Opt-Out. To date, only the 5.4% of people have done so, leaving 95% that can be studied longitudinally with minimal selection.

### Treatment Bias

Studies will need to detail how many patients are treated both before and after the date of inclusion. To date, only 2 studies accounting for 235 patients provided this in full. Ideally, all patients with UIA would be included, and those treated would be censored at the time of treatment, even if this is shortly after recruitment. This ensures a full treatment record and captures all available natural history data.

### Crossover Bias

The rate of treatment on follow-up is generally well reported. The reasons for these treatments are mostly not described. A record of how many patients have follow-up imaging, how many show growth, and how many of those lead to crossing over to treatment is essential.

Although a study with no treatment would be ideal, it is unlikely to be practical. However, it would be beneficial if studies are performed in regions that have relatively less aggressive approaches to UIA and naturally lower treatment rates. Figure 1 would suggest if treatment occurs in up to about 25% that rupture risk estimates will be reasonably representative.

### Follow-Up Bias

Loss to follow-up is a particular concern for this condition, because rupture may result in death or severe disability. Consequently, those lost may be more likely to have had a rupture. Most studies are not clear about how many patients were lost. Most state the duration of follow-up but are unclear if follow-up ended because the study ended, or patients were lost. Given the need for long-term surveillance and the fact that rupture frequently occurs far from the initial treating center, a system that enables comprehensive tracking regardless of patient location is critical. It must also do so irrespective of whether they are answering the phone, mail, or email. The United Kingdom is well positioned for this, with a national database of all hospital admissions and deaths and geographically defined borders. Furthermore, patients leaving the United Kingdom are deregistered from the health system and can be censored. Even those rupturing abroad are usually captured when repatriated or when deaths are recorded on return. This infrastructure supports long-term follow-up with minimal bias.

### SAH Ascertainment

There are notable differences in death rates from rupture, ranging 27% to 65% (Murayama 27%, UCAS 35%, Juvela 53%, ISUIA 65%, Ishibashi, SUAVe, and Wermer not reported). It is difficult to explain such variation, raising questions about how consistently out-of-hospital deaths are recorded. Systems to capture out-of-hospital deaths and countries with high postmortem rates are needed.

### Length of Follow-Up

National databases also provide a solution to the issue of short-term follow-up. An ideal study would continue follow-up indefinitely. This is not realistic within the framework of most research funders but can be delivered utilizing national databases, which are continuously and prospectively updated.

### New Versus Existing Diagnosis

Another way to consider the effect of time on UIA rupture risk is to compare patients with newly diagnosed UIA with those with existing diagnoses. The ratio of these groups within a cohort will also influence the overall rupture rate if rupture risk varies with time from diagnosis. Many studies are unclear whether they have limited patients to those with new diagnoses, or have included patients with existing diagnoses, and none describe whether these groups have different risks. Future studies should collect this data to determine if a newly diagnosed UIA carries a different risk from one that has been known for many years.

### Personalization

Low event rates and limited covariates prevent fine risk stratification. Most studies only consider UIA size and location. Even PHASES used just 5 variables relevant to non-Finnish/Japanese patients. Future studies should include a broader range of baseline predictors of UIA rupture, like family history of IA or SAH, and UIA morphology. Genetics is expected to play a role in IA rupture as well as formation. ROAR-DNA is therefore currently approaching patients in the ROAR study to obtain saliva samples for DNA analysis. Baseline imaging offers further insight into morphology and hemodynamics and is under investigation in ROAR-FLOW. In addition, there is a need for time-varying factors like smoking and blood pressure to be obtained from primary care records, as is currently planned in ROAR-GP. These may be particularly valuable in studying the effects of different medications as well as female hormones (hormone replacement therapy and oral contraceptive pill).

### Sample Size

Studying large numbers of predictors when event rates are so low requires large cohorts. No study to date, other than the PHASES pooled analysis, has been large enough to consider more than a few predictors of UIA rupture. Larger sample sizes will depend on multicenter collaboration. ROAR has recruited 20 445 patients with UIA working with all British Neurovascular centers. Long-term follow-up at this scale is challenging. In ROAR, this is made possible using national databases of hospital admissions and deaths.

## Conclusions

In the absence of randomized controlled trial-level data, treatment decisions for UIA depend on our understanding of their natural history. Guidelines do not make explicit recommendations on which data to use. The American Heart Association and American Stroke Association state that ISUIA and UCAS “are the most carefully designed studies,” but do not reference PHASES. The European Stroke Organisation present their own pooled analysis but provides no individualized risk estimates.

Therefore, clinicians outside Finland and Japan face a dilemma: use the most representative data set,^8^ the largest data set,^9^ or the only data set with long-term follow-up and no treatment.^25^ Our view is that the most balanced representation comes from PHASES, which gives weight to all available studies, accepting that there is no objective way to prioritize their results. However, it remains subject to the limitations of the underlying data sets, including issues with study populations, selection bias, treatment bias, follow-up bias, and short follow-up times, which are often inherent to the condition.

Addressing these gaps requires larger data sets with long-term follow-up, as well as more patient and UIA detail. The logistics of this may be prohibitive using conventional study designs and demand scalable, innovative data collection. Hopefully, new studies, such as the ROAR study,^16^ the China Intracranial Aneurysm Project,^43^ and the UCAN project^44^ will achieve this and provide what is needed—an assessment of the validity of PHASES, longer-term rupture risk data, and further personalization of UIA rupture prediction models.

## Article Information

### Disclosures

S. Hall reports grants from the National Institute for Health Research. Dr Gaastra reports grants from the Medical Research Council. D. Bulters reports grants from Rosetrees Trust; grants from BPL; grants from UK Medical Research Council; and grants from National Institute for Health Research. The other authors report no conflicts.

### Supplemental Material

Tables S1–S5

Figure S1

## APPENDIX

ROAR Study Group

Brighton—Giles Critchley, Ben Fisher; Birmingham—Jonathan Thant, Ed White; Bristol—Mario Teo; Cambridge—Adel Helmy; Cardiff—James Galea, Janneke van Beijnum; Dundee—David Bennett; Edinburgh—Paul Brennan, Anthony Wiggins, Jonny Downer; Glasgow—Jerome St George; Hull—Gueorgui Kounin; London (Barts)—Chris Uff; London (Imperial)—Ramesh Nair, Kyriakos Lobotesis; London (Kings)—Christos Tolias; London (Queen Square)—Patrick Grover; London (St George’s)—Pawan Minhas; Leeds—Ian Anderson; Liverpool—Emmanuel Chavredakis; Manchester—Hiren Patel; Middlesborough—Nitin Mukherji; Newcastle—Patrick Mitchell, Nick Ross; Nottingham—Graham Dow; Oxford—Jash Patel, Samir Matloob; Plymouth—Peter Whitfield; Preston—Nihal Gurusinghe; Romford—Raghu Vindlacheruvu; Sheffield—Andrew Bacon; Stoke—Nikolaos Tzerakis

## Supplementary Material
