# Supplementary material for: Estimating Rupture Risk of Intracranial Aneurysms: What We Know, What We Do Not Know, and What We Need
Source: Stroke. 2026 Apr 8;57(6):1821–33. doi: 10.1161/STROKEAHA.125.054863 (PMC13196851; doi:10.1161/STROKEAHA.125.054863)
Supplement: Supplementary file 1 [file str-57-1821-s001.pdf]

Supplemental Materials

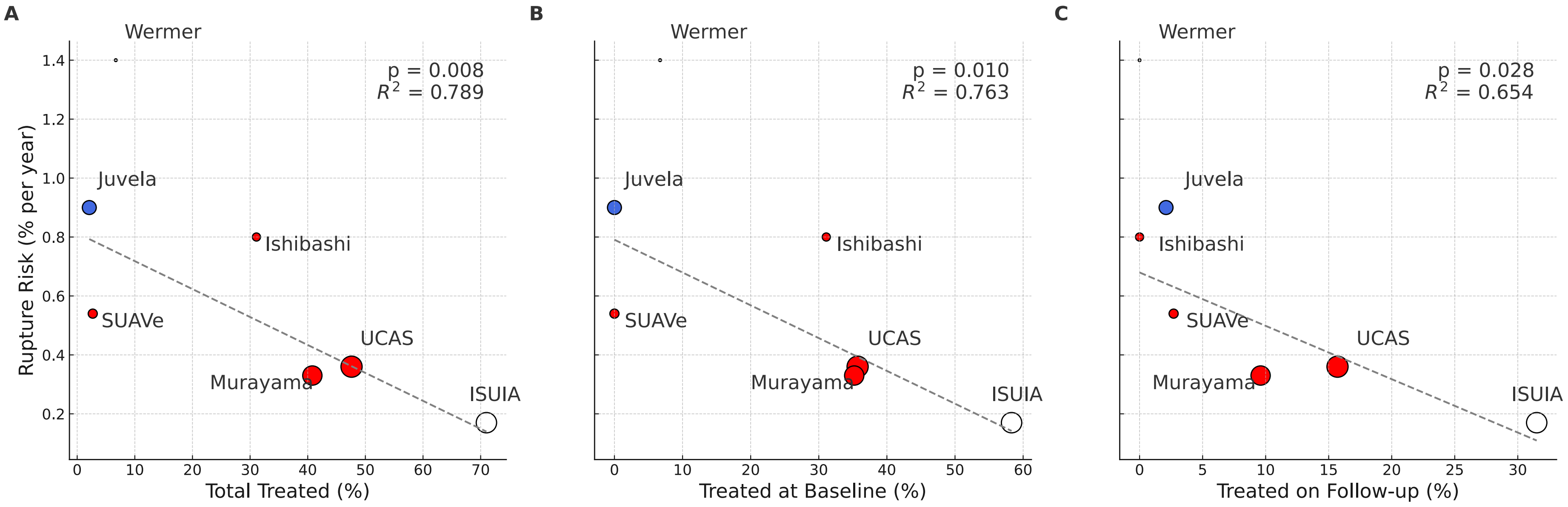

Figure S1 The relationship of observed rupture risk for small UIA with treatment rates in studies*. A shows all treatments, and B and C divide this into treatment rate at baseline and treatment rate on follow-up, with both showing similar patterns. Size of points is scaled to study size (number of patient-years of follow-up). Finnish studies are in blue, Japanese in red and Non-Finnish Non-Japanese in white. Grey dotted line is weighted linear regression line (weighted by study size).*

**AHA/ASA guidelines**	**ESO guidelines**
Prior history of aSAH may be considered to be an independent risk factor for future hemorrhage secondary to a different small unruptured aneurysm<br><br>*Size of effect: Class IIb*<br>*Estimate of certainty: Level of Evidence B*	In adult patients in whom the estimated 5-year risk of aneurysm rupture is higher than the risk of the preventive treatment modality, we suggest preventive aneurysm repair with the treatment modality that is most effective and safe for that particular aneurysm.<br><br>*Quality of evidence: Very low*<br>*Strength of recommendation: Weak for intervention*
Patients with aneurysms with documented enlargement during follow-up should be offered treatment in the absence of prohibitive comorbidities<br><br>*Size of effect: Class I*<br>*Estimate of certainty: Level of Evidence B*	In adult patients with growth of a UIA detected at follow up imaging, we suggest preventive aneurysm repair. However, despite an increased risk of rupture in such patients, this risk remains to be weighed against the risk of treatment complications.<br><br>*Quality of evidence: Very low*<br>*Strength of recommendation: Weak for intervention*
Treatment of UIAs in patients with a family history of IA is reasonable even in aneurysms at smaller sizes than spontaneously occurring IAs<br><br>*Size of effect: Class IIa*<br>*Estimate of certainty: Level of Evidence B*	For adult patients with UlA we suggest that the recommendation for versus against preventive aneurysm repair by the multidisciplinary team should be based on:<br>• aneurysm-related risk factors for rupture, that is, UlA size, location and lobulation<br>• risk factors for rupture, that is, previous SAH from a different aneurysm, family history for UIA or SAH, smoking and hypertension<br>• UIA growth (1 mm in any diameter) or de novo formation on serial imaging<br>• life expectancy<br>• risk factors for treatment complications, that is, patient age and comorbid disease, aneurysm morphology and complexity and estimated risk of treatment).<br><br>*Expert Consensus*
Several factors, including patient age and aneurysm location and size, should be taken into account when considering surgical clipping as the mode of treatment for a UIA<br><br>*Size of effect: Class I*<br>*Estimate of certainty: Level of Evidence B*	In asymptomatic adult UlA patients with significant comorbid diseases and/or reduced life expectancy (<5 years), we suggest no preventive aneurysm repair.<br><br>*Expert consensus*

Table S1 main recommendations from AHA/ASA guidelines regarding natural history of UIA and preventive repair

PubMed search string

#1:
"intracranial aneurysm"[Title/Abstract] OR "intracranial saccular aneurysm"[Title/Abstract] OR "cerebral aneurysm"[Title/Abstract] OR "intracranial aneurysms"[Title/Abstract] OR "intracranial saccular aneurysms"[Title/Abstract] OR "cerebral aneurysms"[Title/Abstract]

#2:
"risk of rupture"[Title/Abstract] OR "aneurysm rupture"[Title/Abstract] OR "risk factors"[Title/Abstract] OR "rupture"[Title/Abstract] OR "unruptured"[Title/Abstract] OR "subarachnoid hemorrhage"[Title/Abstract]

#3:
"follow-up"[Title/Abstract] OR "follow up"[Title/Abstract] OR "natural history"[Title/Abstract] OR "natural course"[Title/Abstract]

#1 AND **#2** AND **#3**

Table S2 Search terms used for review of the literature post-PHASES
PubMed was searched from 26/7/13 to 11/6/25.
Embase was not used.

Author<br>Recruitment period	Country	Inclusion criteria	Number of patients	Mean age (years)	Mean Follow-up (years)	Rupture events
Guresir<br>1999-2012	Germany	Anterior circulation, <7mm, no family or personal history of SAH	263	55	4.0	3
Kubo<br>2006-2013	Japan	>2mm UIA, age >70 years	79	74	3.2	0
Murayama<br>2003-2012	Japan	Saccular, non-cavernous, >2mm	2,252	65	2.6	56
Teo<br>2006-2012	UK	UIA	94	53	3.4	4
Molenberg<br>1998-2017	Netherlands	Age >18, intra-dural	206	56	1.0*	1
Rinaldo<br>1997-2015	USA	Age >65 years	214	75	3.7*	8
Weng<br>2016-2019	China	UIA 2-7mm, ICVD, 18-80 years, no family or personal history of SAH	315	59	1.4	0
Han<br>1993-2010	Korea	De novo aneurysms	63	48	5.0*	18
Hurford<br>2011-2020	UK	TIA	95	70	4.5	1

Table S3: Post-PHASES natural history studies of patients with UIA.
**median*

Study	Definition of small	Total treated	Treated at baseline	Treated on follow-up	Aneurysm rupture risk *(rupture/year)*	Extrapolated rupture risk *(rupture/30 yrs)*
ISUIA	<7mm	71.5%*	58.3%	31.6%	0.17%[§]	4.8%
UCAS	<5mm	47.6%*	35.7%	15.7%	0.36%	11.4%
Wermer	<5mm	6.7%	6.7%	0%	1.40%[‖]	34.4%
SUAVe	<5mm	2.7%	0%	2.7%	0.54%	15.0%
Ishibashi	<5mm	31.4%[†]	31.4%	0%	0.80%	21.4%
Juvela	2-6mm	2.1%[‡]	0%	2.1%	0.90%	23.8%
Murayama	<5mm	40.8%*	35.2%	9.6%	0.33%	9.4%

Table S4: Treatment rates and observed rupture risk for small UIA. This includes all locations (published data does not allow separation by anterior or posterior circulation). Studies included are those in the PHASES analysis or subsequent studies with >2,000 years follow-up. Aneurysm rupture risk is expressed as the mean risk of rupture per year over the duration of study follow-up.

**includes treatments performed at inclusion and those on follow-up. [†] Data stated as not available in manuscript, but derived from text in PHASES. [‡]3 treatments occurred 24-26 years after diagnosis. [§]type 1 and 2 patients (with and without prior SAH) combined. [‖]based on rupture of a previously treated aneurysm.*

Type of Bias		ISUIA	Wermer	Ishibashi	SUAVe	UCAS	Juvela	Murayama	ROAR
Selection Bias	Sampling Bias	Likely High<br><br>many treated outside of study	Low<br><br>all patients selected for screening after SAH or for familial aneurysms included	Likely Low<br><br>presumed to include all patients seen in a single neurosurgical centre	Unknown	Unknown<br><br>discussion states it is low, but 59% of institutions recruited <10 cases	None<br><br>all patients having angiograms included	Likely Low<br><br>presumed to include all patients seen in a single neurosurgical centre	None
	Referral Bias	High<br><br>many quarternary referral centres	High<br><br>only patients referred for screening	Unknown	Unknown	Unknown	High<br><br>only patients having DSA	Unknown	Moderate<br><br>all patients undergoing CTA or MRA
	Volunteer Bias	High	High	Low	Unknown	Unknown	Low	Low	Low<br><br>Excludes only 5% of patients who have opted out of digital research
	Prevalent Case Bias	Possibly high<br><br>does not state if those with existing diagnosis excluded	None	Possibly high<br><br>does not state if those with existing diagnosis excluded	Possibly high<br><br>does not state if those with existing diagnosis excluded	None	None	Possibly high<br><br>does not state if those with existing diagnosis excluded	None<br><br>analyses patients with existing diagnosis as a separate group
	Healthy User Bias	High<br><br>participant in studies less likely to smoke or have untreated hypertension	High<br><br>participant in studies less likely to smoke or have untreated hypertension	Low	High<br><br>participant in studies less likely to smoke or have untreated hypertension	High<br><br>participant in studies less likely to smoke or have untreated hypertension	Low	Low	Low
	Berkson's Bias	High<br><br>many aneurysms likely to have been diagnosed due to scans	Low	High<br><br>many aneurysms likely to have been diagnosed due to scans	High<br><br>many aneurysms likely to have been diagnosed due to scans	High<br><br>many aneurysms likely to have been diagnosed due to scans	High<br><br>more likely to smoke or have hypertension if had SAH	High<br><br>many aneurysms likely to have been diagnosed due to scans	High<br><br>many aneurysms likely to have been diagnosed due to scans

		done for other health problems		done for other health problems	done for other health problems	done for other health problems		done for other health problems	done for other health problems
Treatment Bias	Confounding by Indication	High<br><br>58% treated at baseline	Low<br><br>7% treated at baseline	Moderate<br><br>31% treated at baseline	Low<br><br>0% treated at baseline	Moderate<br><br>36% treated at baseline	Low<br><br>0% treated at baseline	Moderate<br><br>35% treated at baseline	Low<br><br>19% treated at baseline
	Crossover Bias	High<br><br>32% treated on follow up	Low<br><br>0%	Low<br><br>0%	Low<br><br>3%	Moderate<br><br>16%	Low<br><br>2%	Low<br><br>10%	Low<br><br>8%
	Performance Bias	Moderate<br><br>ad hoc imaging as clinically indicated, number performed not available	High<br><br>scheduled follow-up scans for all patients	Moderate<br><br>ad hoc imaging as clinically indicated, number performed not available	High<br><br>scheduled follow-up scans for all patients	Moderate<br><br>ad hoc imaging as clinically indicated, number performed not available	Moderate<br><br>ad hoc imaging as clinically indicated, number performed not available	Moderate<br><br>ad hoc imaging as clinically indicated, number performed not available	Moderate<br><br>ad hoc imaging as clinically indicated, number of scans performed recorded
Information Bias	Misclassification Bias	Moderate<br><br>all SAH verified on CT, LP or post mortem, but 36 patients who had both an aneurysm and another potential source of subarachnoid haemorrhage were excluded from analysis	Low<br><br>SAH verified on CT	Unknown	Unknown	Low<br><br>all SAH verified on CT, LP or post mortem	Low<br><br>interview of post mortem or death certificate	Unknown	Low<br><br>all SAH verified on CT, LP, post mortem or death certificate
	Observer Bias	Low	Low	Low	Low	Low	Low	Low	Low
	Recall Bias	Low	Low	Low	Low	Low	Low	Low	Low
	Detection Bias	Low	Low	Low	Low	Low	Low	Low	Low
	Attrition Bias	Low<br><br>0.2% lost to follow-up	Low<br><br>4% lost to follow-up	Unknown	Moderate<br><br>14% lost to follow-up	High<br><br>22% lost to follow-up	None<br><br>0% lost to follow-up	Low<br><br>2% lost to follow-up	None<br><br>0% lost to follow-up
	Ascertainment Bias	Low	Low	Low	Low	Low	Moderate	Low	Low

							Large part of the study undertaken prior to CT diagnosis of SAH		
Confounding	Confounding Variables	High<br><br>eg smoking, location, treatment rate	High<br><br>eg smoking, location, treatment rate	High<br><br>eg smoking, location, treatment rate	High<br><br>eg smoking, location, treatment rate	High<br><br>eg smoking, location, treatment rate	High<br><br>eg smoking, location, treatment rate	High<br><br>eg smoking, location, treatment rate	High<br><br>eg smoking, location, treatment rate
	Residual Confounding	High<br><br>Did not account for aneurysm shape in primary analysis	NA<br><br>Insufficient events	High<br><br>Did not account for aneurysm shape in primary analysis	High<br><br>Did not account for aneurysm shape in primary analysis	Low<br><br>Multivariable model accounts for shape and three levels of smoking	High<br><br>Did not account for aneurysm shape in primary analysis	Low<br><br>Multivariable model accounts for shape and three levels of smoking	Low<br><br>Multivariable model accounts for shape and three levels of smoking
	Time-varying Confounding	High<br><br>Changes in smoking or blood pressure not accounted for	High<br><br>Changes in smoking or blood pressure not accounted for	High<br><br>Changes in smoking or blood pressure not accounted for	High<br><br>Changes in smoking or blood pressure not accounted for	High<br><br>Changes in smoking or blood pressure not accounted for	Moderate<br><br>Changes in smoking recorded and binaries as quite before or after follow-up	High<br><br>Changes in smoking or blood pressure not accounted for	Low<br><br>Collecting serial blood pressure measurements and smoking status
Analytic bias	Overfitting	Unknown<br><br>No internal or external validation performed	High<br><br>Only one rupture event but analyse multiple variables	Unknown<br><br>No internal or external validation performed	Unknown<br><br>No internal or external validation performed	Unknown<br><br>No internal or external validation performed	Unknown<br><br>No internal or external validation performed	Unknown<br><br>No internal or external validation performed	Low<br><br>Internal validation planned and external dataset being collected
	Model Misspecification	High<br><br>Single level model for multilevel hierarchical problem	High<br><br>Single level model for multilevel hierarchical problem	High<br><br>Single level model for multilevel hierarchical problem	High<br><br>Single level model for multilevel hierarchical problem	High<br><br>Single level model for multilevel hierarchical problem	High<br><br>Single level model for multilevel hierarchical problem	High<br><br>Single level model for multilevel hierarchical problem	Low<br><br>Multilevel models planned
	Survivorship Bias	Possibly high<br><br>does not state if those with	None	Possibly high<br><br>does not state if those with	Possibly high<br><br>does not state if those with	None	None	Possibly high<br><br>does not state if those with	None<br><br>analyses patients with

		existing diagnosis excluded		existing diagnosis excluded	existing diagnosis excluded			existing diagnosis excluded	existing diagnosis as a separate group
	Immortal Time Bias	None	None	None	None	None	None	None	None
	Publication Bias	Low<br><br>Prospectively planned multicentre study	Moderate	High<br><br>Single centre series	Low<br><br>Prospectively planned multicentre study	Low<br><br>Prospectively planned multicentre study	Moderate	High<br><br>Single centre series	Low<br><br>Prospectively planned multicentre study

Table S5: An assessment of potential biases in existing studies *including assessment how the Risk Of Aneurysm Rupture study can address these. Red = High risk, Orange = Moderate risk, Green = Low risk, Grey = Unknown.*
